# *Boesenbergia rotunda* displayed anti-inflammatory, antioxidant and anti-apoptotic efficacy in doxorubicin‐induced cardiotoxicity in rats

**DOI:** 10.1038/s41598-023-38560-5

**Published:** 2023-07-14

**Authors:** Linye Zhang, Qihong Jiang, Xiuming Wang, Amit Jaisi, Opeyemi Joshua Olatunji

**Affiliations:** 1 The Second Peoples Hospital of Wuhu, Wuhu City, 241001 Anhui China; 2 School of Pharmacy, Walailak University, Thasala, 80160 Nakhon Si Thammarat Thailand; 3 African Genome Center, Mohammed VI Polytechnic University, 43150 Ben Guerir, Morocco; 4 Traditional Thai Medical Research and Innovation Center, Faculty of Traditional Thai Medicine, Prince of Songkla University, Hat Yai, 90110 Songkhla Thailand

**Keywords:** Biochemistry, Drug discovery, Medical research

## Abstract

This study evaluated the cardioprotective properties of *Boesenbergia rotunda* extract (BrE) against doxorubicin (DOX) induced cardiotoxicity. Rats received oral gavage of BrE for 28 days and DOX (5 mg/kg/week for 3 weeks). Thereafter the animals were sacrificed, blood and cardiac samples were collected for biochemical, histological and immunohistochemical analyses. The results indicated that BrE attenuated DOX triggered body and cardiac weight loss and prevented against cardiac injury by mitigating histopathological alterations in cardiac tissues as well as serum cardiac function enzymes. BrE significantly reduced serum levels of aspartate transaminase (AST), alkaline phosphatase (ALP), lactate dehydrogenase (LDH), troponin T (TnT) and creatine kinase-MB (CK-MB) in DOX-treated rats. Furthermore, BrE alleviated cardiotoxicity by reducing DOX instigated oxidative stress and potentiating the level of glutathione, as well as the activities superoxide dismutase and catalase in cardiac tissues. In addition, BrE significantly decreased the characteristic indices of DOX-induced cardiac inflammation and apoptosis. Immuno-histochemical analysis revealed that BrE decreased the stain intensity of p53 and myeloperoxidase (MPO) proteins compared to the DXB alone group. In conclusion, our results indicated that BrE modulated oxidative stress, inflammation and apoptosis to attenuate DOX-induced cardiac damage.

## Introduction

Doxorubicin (DOX) is a widely used antitumor agent with broad spectrum application for the management of various cancers. Since the discovery of DOX, it has been shown to be efficacious against solid tumors, Hodgkin’s disease, Kaposi’s sarcoma, leukaemia, breast and lung cancers and lymphomas^1,2^. Unfortunately, the application of DOX in clinical practice is broadly curtailed because of its prevailing cardiac toxicity^1,3^. DOX triggered cardiac toxicity involves several mechanisms, including oxidative stress, redox cycling, excessive production of ROS, apoptosis, mitochondrial impairment and inflammatory reactions^4–6^. In addition, DOX instigated ROS and oxidative imbalance is magnified through the reduction of cardiac antioxidative enzymes activities as well as ensuing increase in pro-inflammatory mediators, leading to DOX-triggered cardiomyopathy^6–8^. Recently, several novel pharmacological approaches have been employed in counteracting cardiotoxicity. For instance empaglifozin, a SGLT-2 inhibitor was shown to offer cardio-protection via inhibiting lipid peroxidation (8-iso prostaglandin f2α), inflammation, apoptosis, ferroptosis and fibrosis in DOX treated mice^9^. As such, therapeutic strategies that target ROS production, oxidative stress and inflammation may be beneficial in mitigating DOX-triggered cardiac toxicity^10–12^.

Medicinal plants are rich reservoir of antioxidant compounds including flavonoids, polyphenols, stilbenes and anthocyanins, with numerous biological properties. These compounds confer prominent anti-inflammatory and antioxidant activities displayed by these plants, which enhances their marked efficacy against several pathological conditions including cancer, hypertension, diabetes and cardio-metabolic disorders^13,14^. *Boesenbergia rotunda* (also known as fingerroot) is a culinary and medicinal plant from the family Zingiberaceae. It is very famous in several southeast Asian countries and China due to its multiple pharmacological effects and appetite promoting properties^15,16^. As previously reported, *B. rotunda* has prominent anticancer, antioxidant, anti-inflammatory, anti-apoptotic wound healing, antiulcer, antidiabetic and antibacterial effects^16–19^. *B. rotunda* polyphenolics especially flavonoids and chalcone derivatives are the major bioactive compounds in the plant accounting for the reported bioactivity of the plant^15,20,21^. Regarding the antioxidant and anti-inflammatory properties of *B. rotunda*, it has been shown that *B. rotunda* suppressed lipid peroxidation, protein kinase B (Akt) and nuclear factor kappa-light-chain-enhancer of activated B cells (NF-KB), while simultaneously increasing antioxidant defense^20,21^. However, the efficacy of *B. rotunda* against DOX-induced cardiac toxicity has not been explored. Since the pathogenies of DOX induced toxicity has been linked to oxidative stress and inflammatory reactions, and considering the excellent antioxidant and anti-inflammatory effects of *B. rotunda*, we envisaged that it may have potentials in mitigating cardiac toxicity triggered by DOX. Herein, this study explored the cardiac-protective properties of *B. rotunda* against DOX-elicited cardiotoxicity in rats.

## Materials and methods

### Plant material

The collection, identification and extraction protocol for *B. rotunda* was detailed in our earlier report^20^. Briefly, the dried and powdered rhizomes of *B. rotunda* was macerated in in 5 L of 95% ethanol for 24 h. The ethanolic solution obtained after filtration was evaporated under reduced pressure via rotary evaporator. Subsequently, the ethanolic extract was extracted with hexane and ethyl acetate. The ethyl acetate fraction (BrE) was dried and refrigerated until further use. All plant experiments were conducted in accordance to relevant institutional, national, and international guidelines and legislation.

### Animal experiment

The protocols used for the experimental animal studies was approved by the committee in charge of animal ethics of The Second Peoples Hospital of Wuhu (WHEYLLWYH-2021-0928). In addition, all animal experimental protocols adhered to the guidelines of the National Institutes of Health Guide for the Care and Use of Laboratory animals (NIH Publications No. 8023, revised 1978). The current study also adheres to the ARRIVE Guidelines for reporting in vivo experiments. Seven weeks old adult male SD rats (160 ± 30 g) obtained from Tianqin Biotechnology, Changsha, China were acclimatized for 7 days and given unrestricted access to food and water and were kept in a facility with standard environmental conditions. Upon completion of adaption, the rats were randomly allotted into groups of six rats per group as follows:

1. Normal control group: healthy rats without any intervention which received 5% DMSO as vehicle once daily for 28 days.
2. BrE control group: healthy rats without any intervention which received BrE (400 mg/kg) once daily for 28 days.
3. DOX control group: rats which received 5% DMSO once daily for 28 days and DOX (5 mg/kg, i.p.) on days 7, 14 and 21.
4. BrE100 + DOX group: rats received BrE (100 mg/kg) once daily and DOX (5 mg/kg, i.p.) on days 7, 14 and 21.
5. BrE400 + DOX: BrE400 + DOX group: rats received BrE (400 mg/kg) once daily and DOX (5 mg/kg, i.p.) on days 7, 14 and 21.

BrE was dissolved in 5% DMSO and the rats received intraperitoneal injections of DOX (5 mg/kg/week) for 3 weeks. The BrE treatment groups were pre-treated with BrE (100 and 400 mg/kg/day) by gavage, and rats were then intraperitoneal injected with DOX. The concentrations of BrE and DOX as well as the time of treatment used in this study were referred to previous report^1,20,22^. The body weight of the animals was periodically measured using a weighing balance during the study.

### Animal sacrifice and biochemical analysis

At the end of treatment, the rats were anaesthetised with sodium thiopental (100 mg/kg) via intraperitoneal injection, followed by blood collection through cardiac puncture. The clotted blood samples were centrifuged to obtain the serum which was further used for assessing cardiac biochemical parameters including aspartate transaminase, alkaline phosphatase, lactate dehydrogenase, troponin T and creatine kinase-MB using diagnostic kits (Thermo Fisher Scientific, Waltham, MA, USA) on a semi autoanalyzer (Dirui CS 600B autochemistry analyzer, Japan) or by ELISA assay kit from Nanjing Jiancheng Bioengineering Institute, China, following the manufacturer's instructions. The cardiac tissues were harvested, rinsed with physiological saline solution, weighed and thereafter homogenised in sodium phosphate buffer (pH 7.4), centrifuged and the supernatants were used for subsequent assays.

### Nitro-oxido-inflammatory parameters assay

Cardiac nitro-oxido-inflammatory parameters including nitric oxide (NO), superoxide dismutase (SOD), catalase (CAT), glutathione (GSH), malonaldehyde (MDA), interleukins 6 and 1β (IL-6 and IL-1β) and tumor necrosis factor alpha (TNF-α), caspase 3 and nuclear factor kappa B (NF-кB) were determined in the tissues’ homogenates with related ELISA detection kits (Abcam, UK), Jiancheng Bioengineering Institute (Nanjing, Jiangsu, China) and Cusabio Technology, China.

### Histopathology

Histological analysis was performed using routine hematoxylin and eosin (H&E) procedures according to previous report^20^. The degree of cardiac tissue damage was assessed with the aid of a semiquantitative scoring assay on four random fields of each histopathological sections from the treatment groups. The severity of the alterations in the cardiac histology were scored according to the follows:

- None (−): representing no involvement of the examined field
- Mild (+): representing involvement of 0–25% of the examined field
- Moderate (++): representing involvement of 25–50% of the examined field
- Severe (+++): represented involvements of 50–100% of the examined field

The grading of the average severity is as follows; Grade 1 < 5% = mild; Grade 2 = 16–25% = moderate; Grade 3 > 35% = severe.

### Immunohistochemical staining

Likewise the immunostaining procedures for tumor protein P53 (p53) and myeloperoxidase (MPO) were assessed using routine methods based on the previous study of Xing et al.^1^. The relative expression intensity was analyzed using Image J software.

### Statistical analysis

The results were displayed as mean ± SD and evaluated statistically using GraphPad Prism software 5.0. All parameters were analysed using one-way ANOVA with Tukey’s multiple comparison test. Values were considered significant at *p* < 0.05.

### Ethical approval

The protocols used for the experimental animal studies was ratified by the committee in charge of animal ethics of Second Peoples Hospital of Wuhu City (WHEYLLWYH-2021-0928). In addition, all animal experimental protocols adhered to the guidelines of the National Institutes of Health Guide for the Care and Use of Laboratory animals (NIH Publications No. 8023, revised 1978). The current study also adheres to the ARRIVE Guidelines for reporting in vivo experiments.

## Results

### Effect of BrE on body and cardiac weight

**Figure 1:**
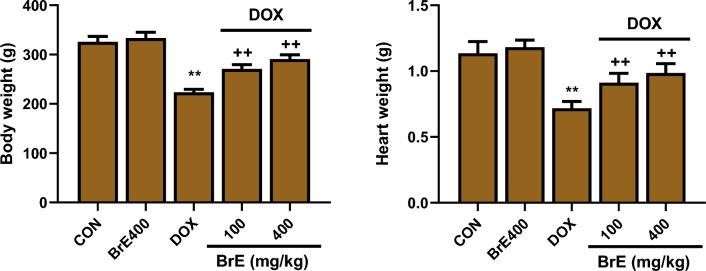
Effect of BrE on body and heart weights in DOX administered rats. Data are expressed as mean ± SD (n = 6). **Denotes significant differences compared to the CON and BrE 400 groups (*p* < 0.05); +  + denotes significant differences compared to the DOX group (*p* < 0.05).

The administration of DOX once weekly for three weeks triggered significant reduction in the body and heart weights of rats in the DOX group when juxtaposed with the normal rats (Fig. 1). Interestingly, BrE dose dependently increased the body gain of rats administered with DOX. It was also observed that the heart weight of BrE treatment groups was significantly increased in contrast to the DOX animals (Fig. 1).

### Effect of BrE on serum biochemical parameters

**Figure 2:**
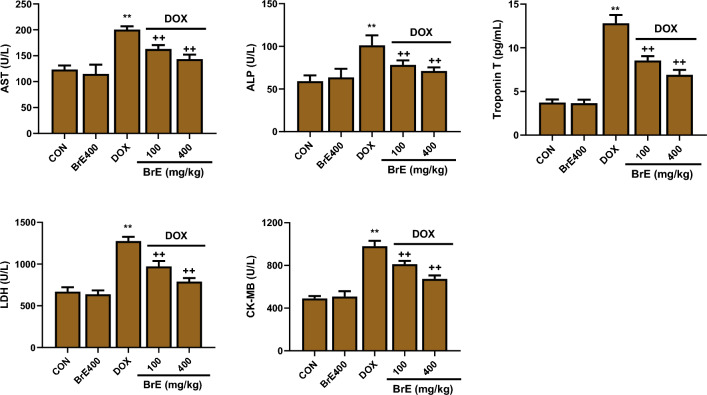
Effect of BrE on serum levels of AST, ALT, Troponin T, LDH and CK-MB in DOX administered rats. Data are expressed as mean ± SD (n = 6). **Denotes significant differences compared to the CON and BRE400 groups (*p* < 0.05); ++Denotes significant differences compared to the DOX group (*p* < 0.05).

The impact of BrE treatment on various biochemical parameters in DOX administered rats is revealed in Fig. 2. The weekly administration of DOX to the rats for 3 successive weeks induced considerable (*p* < 0.05) increases in the serum levels of AST (200.50 ± 6.34 U/L), ALP (101.16 ± 11.78 U/L), LDH (1276.23 ± 50.07 U/L), TnT (12.81 ± 0.94 pg/mL) and CK-MB (979.06 ± 51.42 U/L) compared with the normal and BrE400 control rats. Whereas, pre-treatment and concurrent treatment with either low or high doses BrE exhibited a dose-dependent reduction in the serum concentrations of these markers of cardiac functions compared to the DOX rats. The high dose of BrE (400 mg/kg) showed better protective effects(Fig. 2).

### Effect of BrE on heart histology

**Figure 3:**
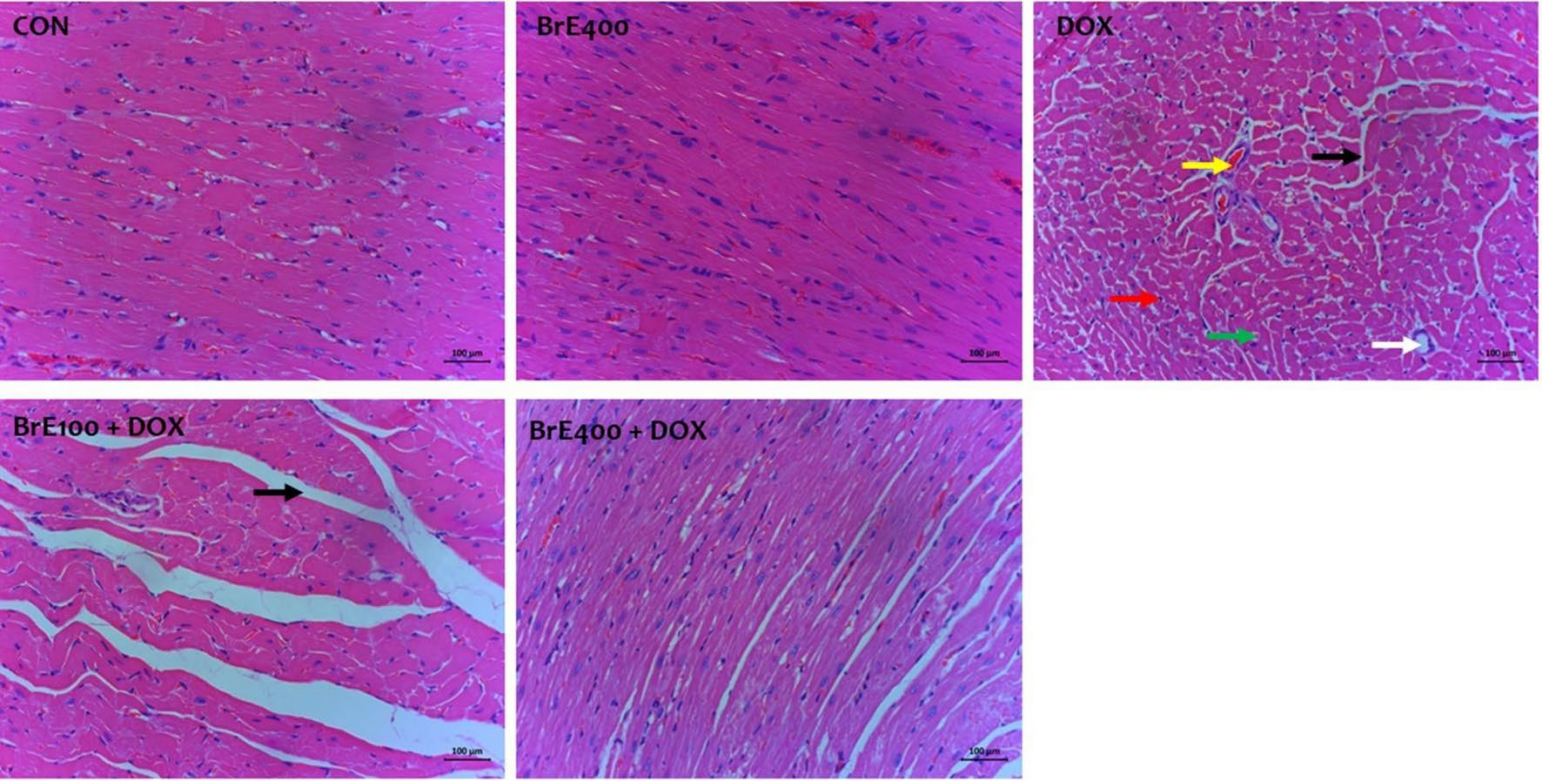
Microscopic pictures of H&E-stained heart sections in of BrE treated rats. Yellow arrow: vascular congestion; Red and green arrow: necrosis cells and pyknotic nuclei; black arrow: degeneration of myocardial fiber, white arrow: vacuolization of sarcoplasm. X: 200; Bar = 100 µm.

Cardiac histopathological analysis of the rats in the normal, DOX and BrE treated groups are depicted in Fig. 3 and Table 1. As observed in Fig. 3A, the cardiac histology of the normal rats showed near perfect cellular and myocardium architecture which was in contrast to the DOX treated only group with vivid pathological alterations including disorganization of the myocardium, loss of myofibrillar and necrotic cells (Fig. 3B). Moreover, noticeable attenuation of the above mentioned alterations induced by DOX were significantly reduced or absent in BrE treated groups (Fig. 3C,D).

**Table 1:** Effect of BrE treatment on histopathologic lesions severity in DOX-treated rats.

Groups	Disorganization	Focal necrosis	Degeneration	Inflammation
CON	0.00 ± 0.00	0.00 ± 0.00	0.00 ± 0.00	0.00 ± 0.00
BrE400	0.00 ± 0.00	0.00 ± 0.00	0.00 ± 0.00	0.00 ± 0.00
DOX	3.00 ± 0.00**	1.80 ± 0.44**	2.20 ± 0.83**	1.40 ± 0.54**
BrE100 + DOX	1.60 ± 0.55^++^	0.90 ± 0.74^++^	1.20 ± 0.57^++^	0.75 ± 0.43^++^
BrE400 + DOX	0.70 ± 0.45^++^	0.20 ± 0.44^++^	0.80 ± 0.27^++^	0.30 ± 0.45^++^

Cardiac injury severity was expressed as mean ± SD (n = 4). **Denotes significant differences compared to the CON and BrE 400 groups (*p* < 0.05); ++Denotes significant differences compared to the DOX group (*p* < 0.05). Grade 1 < 5% = mild. Grade 2 = 16–25% = moderate. Grade 3 > 35% = sever.

### Effect of BrE on p53 and MPO immunohistochemical staining

**Figure 4:**
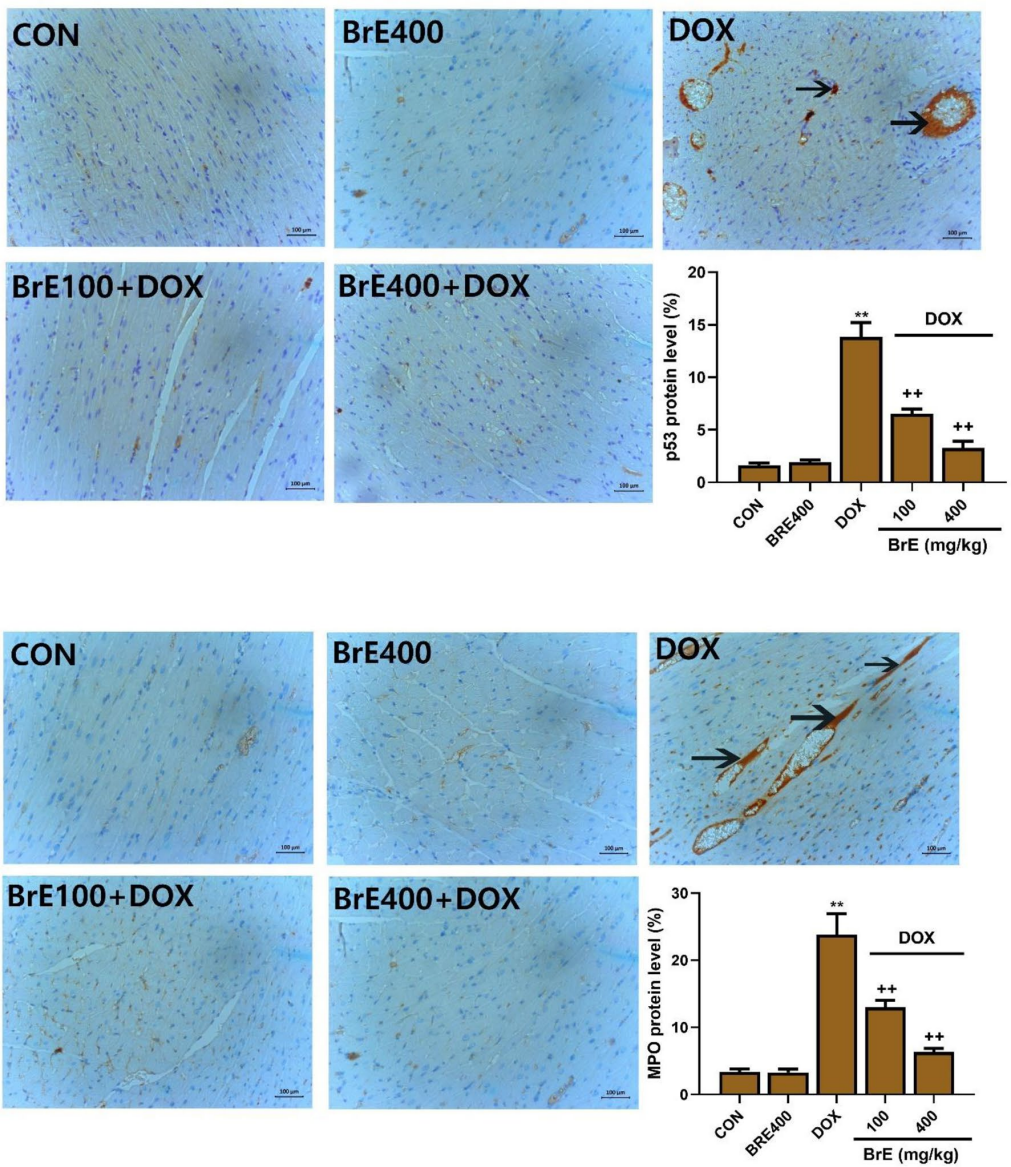
Photomicrograph of heart tissues showing immunohistochemical staining of p53 and MPO. Data are expressed as mean ± SD (n = 6). **Denotes significant differences compared to the CON and BrE400 groups (*p* < 0.05); ++Denotes significant differences compared to the DOX group (*p* < 0.05). X: 100; Bar = 100 µm.

As shown in Fig. 4, the immunochemical staining of cardiac tissues indicated significantly higher number of p53 and MPO positive cells in the DOX treated only group, which was dose dependently decreased by BrE (Fig. 4).

### Effect of BrE on oxidative stress and nitric oxide

**Figure 5:**
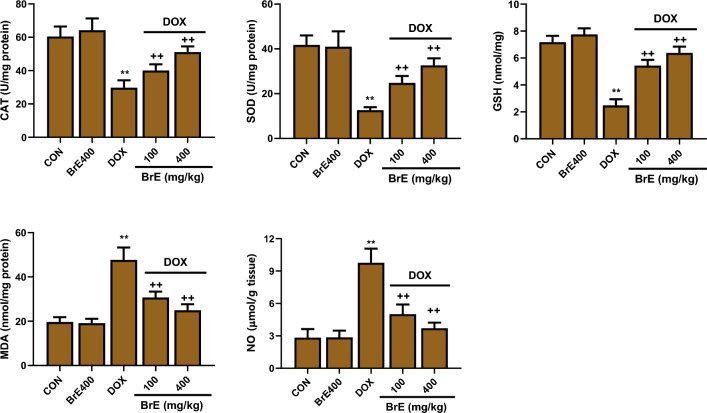
Effect of BrE on cardiac levels of CAT, SOD, GSH and MDA in DOX administered rats. Data are expressed as mean ± SD (n = 6). **Denotes significant differences compared to the CON and BRE400 groups (*p* < 0.05); ++Denotes significant differences compared to the DOX group (*p* < 0.05).

In Fig. 5, the levels of MDA in the DOX treated alone group was notably increased (*p* < 0.05), while cardiac antioxidant enzymes activities: SOD and CAT, as well as GSH level were vividly diminished when juxtaposed with the control rats (Fig. 5). Evidently, BrE supplemented rats dose dependently exhibited remarkably higher cardiac antioxidant enzymes activities, with a reduction in lipid peroxidation (MDA level) when compared to DOX rats (Fig. 5) Furthermore, the DOX group revealed a substantial increase in cardiac NO level when compared to the control groups. In contrast, BrE treatment showed considerable decrease in the cardiac NO level when equated to the DOX group (Fig. 5).

### Effect of BrE on inflammatory markers

**Figure 6:**
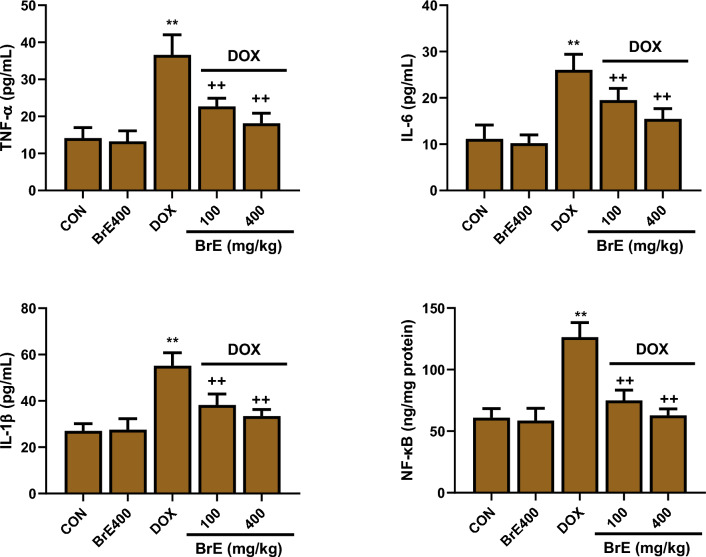
Effects of BrE on cardiac levels of TNF-α, IL-6, IL-1β and NF-кB in DOX administered rats. Data are expressed as mean ± SD (n = 6). **Denotes significant differences compared to the CON and BrE400 groups (*p* < 0.05); ++Denotes significant differences compared to the DOX group (*p* < 0.05).

The levels of cardiac proinflammatory cytokines including TNF-α, IL-1β, IL-6 and NF-кB were significantly elevated in DOX treated only group relative to the normal and BrE control groups (Fig. 6). Whereas the levels of these proinflammatory cytokines were markedly reduced in the DOX administered groups that received both doses of BrE when compared to the DOX group. Interestingly, the low and high doses of BrE (100 and 400 m g/kg) attenuated cardiac TNF-α, IL-1β, IL-6 and NF-кB in a dose-dependent manner (Fig. 6).

### Effect of BrE on caspase 3 activity

**Figure 7:**
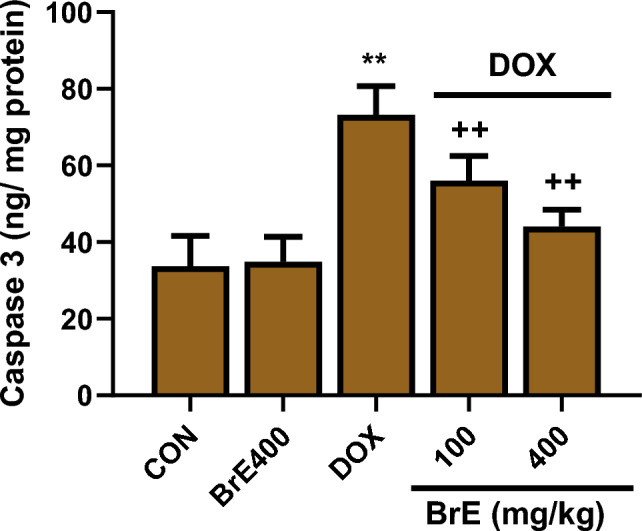
Effects of BrE on cardiac level of caspase 3 in DOX administered rats. Data are expressed as mean ± SD (n = 6). **Denotes significant differences compared to the CON and BrE400 groups (*p* < 0.05); ++Denotes significant differences compared to the DOX group (*p* < 0.05).

Figure 7 represents the effect of BrE on cardiac caspase 3 activities in DOX treated rats. DOX administration led to significant increases in cardiac level of caspase 3 when compared to the control groups (Fig. 7). However, administration of BrE at doses of 100 and 400 mg/kg notably reduced caspase 3 level in a dose dependent manner when compared to the DOX alone treated rats. However, BrE 400 mg/kg dose showed better protective effects (Fig. 7).

## Discussion

*Boesenbergia rotunda* is widely utilized in traditional therapy for the treatment of various disorders, and its protective effects against oxidative organ injury has also been extensively highlighted^23,24^. In our continuous search for alternative treatment for the prevention of chemotherapy induced multi-organ toxicity, we explored the protective effects of *Boesenbergia rotunda* extract (BrE) against cardiotoxicity in rats using doxorubicin as the inducing agent. The results indicated that treatment with BrE ameliorated reduced body weight, preserved cardiac tissue integrity, while decreasing cardiac injury, oxidative stress, inflammation and apoptosis and improving cardiac antioxidant status.

Compared to control animals, there were notable reduction in the body and cardiac weight, which are common features that has been highlighted in cardiotoxicity trigger by DOX^25^. However, treatment with BrE markedly increased the body weight gain as well as the cardiac weight of the treated rats to a level almost comparable to normal group.

Besides, DXB administered rats showed increased serum concentrations of cardiac function biomarkers including LDH, AST, ALP, CK-MB and TnT, suggesting a compromise in cardiac integrity. Under normal physiological circumstances, these biomarkers are found in the cardiomyocytes cytoplasm, however leakages of these enzymes into the blood streams occurs upon cardiomyocytes damage^26,27^. The co-administration of BrE significantly abated the levels of these cardiac biomarkers. Furthermore, the histopathological analysis indicated that BrE offered significant protection of the cardiac tissues, which positively correlated with the reduced serum cardiac function biomarker levels, suggesting the preservation of the myocardium.

It has been extensively documented that DOX can instigate the production of ROS, which subsequently induces oxidative stress and inflammation, two major pathological factors implicated in the development and progression of DOX instigated cardiotoxicity^5,28^. DOX increases the susceptibility of cardiomyocytes to ROS induced oxidative attack through the depletion of antioxidant defense mechanism leading to DNA damage, lipid peroxidation and apoptosis^29–31^. While oxidative stress pathological pathway was activated following DOX administration in this present study as portrayed by increased cardiac MDA and NO levels, as well as substantial decrease in antioxidant enzymes activity, treatment of the animals with BrE significantly decreased cardiac levels of malondialdehyde and nitric oxide with corresponding increase in cardiac glutathione, catalase and superoxide dismutase. Earlier studies have shown that the phytochemical constituents of BrE demonstrated excellent antioxidant ability and their effect on lipid peroxidation, nitric oxide and antioxidant parameters have been highlighted^16,20,23^.

Consistent with the DOX instigated ROS and oxidative stress, the administration of DOX resulted in substantial inflammatory reactions in the cardiac tissues of the rats. Inflammation has been identified as a vital determinant mediating the pathogenesis of DOX triggered cardiotoxicity^6,28,31^. Chemotherapy induced cardiotoxicities have been shown to activate several inflammatory related pathways.

Myeloperoxidase is majorly expressed in multiple inflammatory cells including neutrophils and monocytes, and high levels of MPO has been liked to increased oxido-inflammatory process^32^. Multiple lines of evidences have shown that MPO can instigate the production of excess highly reactive oxidants leading to tissue damages^32,33^. Furthermore, MPO has been linked to the pathogenesis of several cardiovascular disorders^32^. Additionally, earlier reports have also extensively documented that increased production of ROS can trigger the activation of several inflammatory mediators in cardiac tissues^34^. Huge body of evidences have also illustrated that IL-6, IL-1β, TNF-α and NF-кB were elevated in the heart of DOX treated rats, indicating a relationship between inflammation and DOX-cardiotoxicity^34,35^. Moreover, the effect of BrE on inflammation has been reported to be associated with its potentials to supress proinflammatory cytokines^17,20^. In consistent with these previous studies, treatment with BrE markedly reduced MPO, TNF-α, IL-1β, IL-1β and NF-кB levels compared with DOX alone rats.

In addition to oxido-inflammatory mechanism, apoptosis is also considered as a key event mediating DOX instigated cardiac toxicity^36,37^. DOX-induced oxidative stress activates an inherent mitochondrial-dependent apoptotic pathway in cardiac tissues. DOX-induced apoptotic signalling have been shown to involves several processes including the heightening caspase-3, Bax and p53 protein activities^1,36,38,39^. The cardiac expression of p53 protein was performed using immunostaining technique. p53 plays a major role as a tumor suppressor, besides its involvement in several other processes in the body such as aging, cell cycle arrest, apoptosis, DNA damage and oxidative stress^40^. In agreement with other previous studies, this study observed that the administration of DOX gave rise to marked cardiac apoptosis as observed by remarkable increase in caspase 3 and p53 stain intensity in the heart tissues. Notably, BrE treatment significantly ameliorated p53 stain intensity in the treated rats.

Plant derived phytochemicals are promising drug components against several oxidative stress instigated disorders. Our previous phytochemical studies on BrE revealed the presence of bioactive polyphenols: boesenbergin A, pinostrobin, alpinetin pinocembrin, cardamonin and panduratin A^1^. It has been reported that flavonoids are the main components in BrE, and BrE flavonoids are notable for their excellent bioactives, especially as antioxidants, anti-inflammatory and anti-apoptotic agents^1,41–46^. For instance, pinostrobin, inflammatory mediators including nitric oxide, prostaglandin E2, inducible NO synthase, proinflammatory cytokines and nuclear translocation of nuclear factor-κB in LPS primed RAW 264.7 macrophages^47^. Cardamonin, another major bioactive flavone in BrE was reported to protect against LPS-induced cardiac contractile malfunctioning by supressing oxido-inflammation and apoptosis via the Nrf2 and NF-κB pathways^48^. In another study, panduratin A ameliorated cisplatin-induced renal injury via inhibition of extracellular signal-regulated kinase (ERK)1/2, caspase 3 and promoting the expression of anti-apoptotic protein Bcl-2^25^. As such, the flavonoids of BrE may be responsible for the cardioprotective effects against DOX induced toxicity observed in this study.

## Conclusion

This study showed that BrE possesses cardioprotective properties against DOX toxicity through amelioration of oxidative stress, inflammation and apoptosis. These findings thus favours the application of BrE as a counteractive agent against DXB cardiotoxicity. Further studies are required in elucidating the detailed mechanism of actions and the compounds implicated in the cardioprotective effects.

## Data Availability

Data will be available on reasonable request from the corresponding author.
